# Lithium Iron Phosphate/Carbon (LFP/C) Composite Using Nanocellulose as a Reducing Agent and Carbon Source

**DOI:** 10.3390/polym15122628

**Published:** 2023-06-09

**Authors:** Macarena Kroff, Samuel A. Hevia, James N. O’Shea, Izaskun Gil de Muro, Verónica Palomares, Teófilo Rojo, Rodrigo del Río

**Affiliations:** 1 Departamento de Química Inorgánica, Facultad de Química y de Farmacia, Pontificia Universidad Católica de Chile, Santiago 7820244, Chile; makroff@uc.cl; 2 Instituto de Física, Pontificia Universidad Católica de Chile, Santiago 6904411, Chile; sheviaz@uc.cl; 3 Centro Investigación en Nanotecnología y Materiales Avanzados UC (CIEN-UC), Pontificia Universidad Católica de Chile, Santiago 7820244, Chile; 4 School of Physics and Astronomy, University of Nottingham, Nottingham NG7 2RD, UK; j.oshea@nottingham.ac.uk; 5 Departamento de Química Orgánica e Inorgánica, Facultad de Ciencia y Tecnología, Universidad del País Vasco UPV/EHU, 48940 Leioa, Spain; izaskun.gildemuro@ehu.eus (I.G.d.M.); veronica.palomares@ehu.eus (V.P.); teo.rojo@ehu.eus (T.R.); 6 BCMaterials, Parque Científico de la UPV/EHU, 48940 Leioa, Spain

**Keywords:** lithium-ion batteries, lithium iron phosphate (LFP), nanotechnology

## Abstract

Lithium iron phosphate (LiFePO_4_, LFP) is the most promising cathode material for use in safe electric vehicles (EVs), due to its long cycle stability, low cost, and low toxicity, but it suffers from low conductivity and ion diffusion. In this work, we present a simple method to obtain LFP/carbon (LFP/C) composites with different types of NC: cellulose nanocrystal (CNC) and cellulose nanofiber (CNF). Microwave-assisted hydrothermal synthesis was used to obtain LFP with nanocellulose inside the vessel, and the final LFP/C composite was achieved by heating the mixture under a N_2_ atmosphere. The resulting LFP/C indicated that the NC in the reaction medium not only acts as the reducing agent that aqueous iron solutions need (avoiding the use of other chemicals), but also as a stabiliser of the nanoparticles produced in the hydrothermal synthesis, obtaining fewer agglomerated particles compared to synthesis without NC. The sample with the best coating—and, therefore, the best electrochemical response—was the sample with 12.6% carbon derived from CNF in the composite instead of CNC, due to its homogeneous coating. The utilisation of CNF in the reaction medium could be a promising method to obtain LFP/C in a simple, rapid, and low-cost way, avoiding the waste of unnecessary chemicals.

## 1. Introduction

The development of new generations of Li-ion batteries (LIBs) is in constant growth for their use as the energy sources for electric vehicles (EVs) [1,2], as well as for energy storage for sustainable energies, contributing to their intermittence [3,4,5]. Lithium iron phosphate (LiFePO_4_, LFP) cathodes are widely used for these purposes because they have the advantages of low cost, environmental friendliness, thermal stability, and low toxicity, unlike the most widely used cathode material LiCoO_2_ [6]; however, they have the disadvantages of low electric conductivity and poor lithium-ion diffusivity, limiting their use by decreasing their practical capacity [7,8]. Many researchers have studied the diminution of the particle size to improve the diffusion of lithium ions, due to the particle reduction shortening the distances to cover [8,9,10]. Consequently, the synthesis conditions are vital for obtaining a small particle size; hence, there exist many methods to obtain these compounds: solid-state, hydrothermal, solvothermal, etc. [11]. Hydrothermal synthesis is the simplest method to obtain nanoparticles, because it uses simple compounds such as water [12,13]; thus, the use of toxic and harmful chemicals is avoided. Unfortunately, this method faces the difficulty of iron-ion oxidation in aqueous media; hence, it is necessary to include an auxiliary compound that prevents the oxidation. Previous works have shown that hydrothermal synthesis combined with microwave methodology can be used to obtain a homogeneous particle distribution, shorter reaction time, and small particle size, which could be beneficial for cathodes in LIBs [10,14].

On the other hand, to improve the low electric conductivity of LFP, the most widely used method is coating the nanoparticles with carbon compounds. Many different compounds have been studied successfully, including graphene, carbon nanotubes, and more sustainable sources such as glucose and sucrose. Researchers have shown that organic sources of carbon compounds can cover the particles homogeneously [15,16,17], unlike inorganic sources such as carbon nanotubes, whose cylindrical shape makes it difficult for them to cover the nanoparticles completely [18,19,20]. Kanagaraj et al. investigated the incorporation of multi-walled carbon nanotubes (MWCNTs) using an ex situ pathway, obtaining large particles with a lack of electron conduction, leading to a poor specific capacity close to 22 mAhg^−1^ at 0.2 C–rate, using an inorganic compound [21]. Conversely, Meng et al. used in situ coating with an organic source to obtain an LFP material with 1–2 nm of carbon coating and a specific capacity close to 162 mAhg^−1^ at 0.1 C–rate [22].

Therefore, the use of organic sources for the carbon compounds in LFP/C composites seems to be a promising approach that needs to be improved, because one of the main problems is the low quality of the final carbon obtained—since it requires an additional heat treatment [23], in contrast to inorganic compounds—and also, typically, larger particles (attributed to precursors with large dimensions, such as glucose or sucrose [24]) are obtained. For these reasons, it is necessary to search for new materials to obtain LFP/C composites with a smaller particle size.

Nanocellulose (NC), a nanoscale material of cellulose fibre, is the most abundant natural polymer in the world. It can be classified into different types, of which cellulose nanocrystal (CNC) and cellulose nanofiber or nanofibrillated cellulose (CNF) are the most important [25,26]. They differ in the methodology used to obtain them: chemical treatment for the CNC, and delamination of the wood using mechanical procedures in the case of CNF [27,28,29]. Using this kind of material as the carbon source can avoid the use of inorganic, expensive, and toxic compounds such as graphene or carbon nanotubes, and they can cover LFP nanoparticles homogeneously due to their organic nature. NC can also act as the reducing agent [30,31] that iron ions need in hydrothermal synthesis; thus, this nanomaterial is an encouraging compound due to its small particle size in contrast to other organic sources that have been used. Few investigations have used some type of NC to form LFP/C composites. Park et al. [32] reported a mixture of CNF with graphene oxide and LFP that, after heat treatment, formed a composite in which the carbon was produced from the carbonisation of CNF and reduced graphene. This cathode exhibited good performance, with a discharge capacity of 168.9 mAhg^−1^ at 0.1 C–rate, and the researchers attributed these good results to the CNF because it can prevent the GO stacking, act as an extra carbon source, and avoid agglomeration of LFP during annealing. This methodology mixes carbon compounds after the synthesis of LFP—an ex situ pathway. In contrast, in this work, we present a simple methodology using only the reagents for LiFePO_4_, NC, and water as the solvent to obtain the final LFP/C composite—a much more efficient and environmentally friendly methodology that avoids the use of harmful reagents and multiple steps. Moreover, we report an easy method to obtain the LFP/C composite, using NC in the hydrothermal reaction medium as a carbon source, a reducing agent for the iron ions, and a stabiliser to avoid grain size growth, creating a simple and new methodology to create sustainable LFP/C materials. For this purpose, we mixed the precursors of LFP with CNC and CNF separately for further comparisons, in deionised water, and incorporated them into a microwave reactor vessel to heat at different temperatures and for different reaction times. Then, the dry mixture of LFP/NC was carbonised in a tube furnace at 600 °C for 1 h in a N_2_ atmosphere to obtain the LFP/C composite.

## 2. Materials and Methods

Lithium hydroxide (LiOH 98%), iron(II) sulphate heptahydrate (FeSO_4_·7H_2_O), and phosphoric acid (H_3_PO_4_ 85%) were purchased from Merck. CNC (8 wt. %) was obtained from Blue Goose Biorefineries Inc., Canada, and CNF (15% solids) was purchased from the Process Development Center, the University of Maine, USA. As solvents, technical ethanol (EtOH 96%) and Milli-Q water (18.2 MΩcm^−1^) were used for all experiments. Lithium hexafluorophosphate in ethylene carbonate and dimethyl carbonate solution (1.0 M LiPF_6_ in EC/DMC 50/50 *v*/*v*, battery grade) was used as an electrolyte; poly(vinylidene fluoride) Mw ~534,000 (PVDF), carbon Vulcan XC-72C, and 1-methyl-2-pyrrolidinone (NMP) were purchased from Merck and were used to create the electrodes. All reagents were used without further purification.

The synthesis of LFP and its composites was carried out as follows: In a microwave vessel, the precursors for LFP were mixed (LiOH, FeSO_4_·7H_2_O, and H_3_PO_4_, at a molar ratio of 3:1:1) as described in the literature [12,33]. To this mix, we added different weight percentages of CNF and CNC separately for further comparisons, until a homogeneous suspension was formed. First, the LiOH was put into the vessel with ultrapure water until full dissolution, and then H_3_PO_4_ was added to form Li_3_PO_4_. The next step was to incorporate the NC and mix it for a few minutes. Finally, FeSO_4_·7H_2_O was added until full dissolution; ultrapure water and ethanol were then used to fill the total vessel volume (25 mL maximum) and fix the pH of the solution between 6.5 and 8.1, since at this pH a pure LFP phase can be obtained [34,35]. For synthesis without NC, the incorporation of this material in the vessel was omitted. The vessel with the mixed precursor was put into the CE Corporation Discover SP microwave, where 200 W and 17.2 bars were the experimental power and pressure conditions to control the reaction, respectively. Different temperatures and reaction times were selected to analyse the formation of LFP through this route. After the microwave reaction, the mixture was washed and centrifuged several times with deionised water, before being left to dry overnight and obtain LFP or LFP/NC powder. To obtain the LFP/C composites, the powder obtained in the previous step (LFP/NC) was put into a tube furnace for heat treatment at 600 °C for 1 h, with a N_2_ atmosphere (99.9% yield, from Indura, Chile).

Morphological characterisations were carried out with field-emission scanning electron microscopy (FE-SEM) using an FEI microscope (model: Quanta FEG 250) and transmission electron microscopy (Philips CM200) with an accelerating voltage of 200 kV and a point resolution of 0.235 nm. FT-IR spectroscopy was carried out using a Nicolet iS10 (Thermo Scientific) between 4000 and 400 cm^−1^ wavelengths, using KBr pellets at 1:100% wt. Raman spectra were used to analyse the presence of carbon in the created composites using a WITec Alpha 300-RA spectrometer with a 532 nm laser; the D and G bands were deconvoluted with Origin software using Lorentzian curves. XDR diffractograms were measured with a Bruker D8 Advance, with CuKα1 = 1.5604 Å between 2θ angles of 10° and 80°, with 0.20° intervals. Rietveld refinements were analysed using FullProf Suite software. The XPS characterisation was performed with a Devi-sim (SPECS) near-ambient-pressure X-ray photoelectron spectroscopy instrument operating under ultrahigh vacuum, comprising a Phoibos 150-NAP hemispherical analyser and a monochromatic Al Kα X-ray (hυ = 1486.6 eV) source. A flood gun was used for charge compensation. Binding energies were calibrated to the C 1s peak at 284.8 eV; for background subtraction, a Shirley background was used, and for data treatment CasaXPS software was used to fit the spectral curves to pseudo-Voigt lineshapes.

The cathode formation was accomplished as follows: A mix of active material (LFP/C), PVDF, and conductive carbon (Vulcan XC-72) at a weight proportion of 8:1:1 was added to NMP in order to form a homogeneous solution through stirring for 1 h. Then, the slurry was placed onto the aluminium foil with a specific thickness of 100 µm, using the Dr Blade technique. Then, the foil was dried and cut into electrodes of active material of 11 mm in diameter. Coin cell assemblies were prepared inside a glovebox using metallic Li of 13 mm in diameter for the counter and reference electrodes, two drops of electrolyte were used per cell (1.0 M LiPF_6_ in EC/DMC 50/50 *v*/*v*), and polyethylene (PE) was employed as the separator. Cyclic voltammetry was carried out using an Ivium Vertex X.S, between 2.8 and 4.1 V versus Li/Li^+^ at 0.1 mVs^−1^. Charge and discharge curves were measured at a 0.1 C–rate between 2.8 and 4.1 V versus Li/Li^+^ in a Biologic MPG-2 battery cycler.

## 3. Results and Discussion

### 3.1. Synthesis of LFP/C Composites

Table 1 shows the microwave-assisted hydrothermal synthesis conditions investigated for LFP using CNF and CNC as reducing agents and carbon sources. The time and temperature of the reaction were previously investigated for this synthesis using ethanol as a reducing agent (without carbon), as shown in Figure S1. From this analysis, we concluded that 150 °C and 15 min of reaction are the minimum conditions to form LFP using the proposed synthesis method.

The characterisations were performed after heat treatment at 600 °C for one hour in a tube furnace with an inert atmosphere, in order to carbonise the NC into a conductive material and form LFP/C composites, due to NC being a non-conductive material.

**Figure 1:**
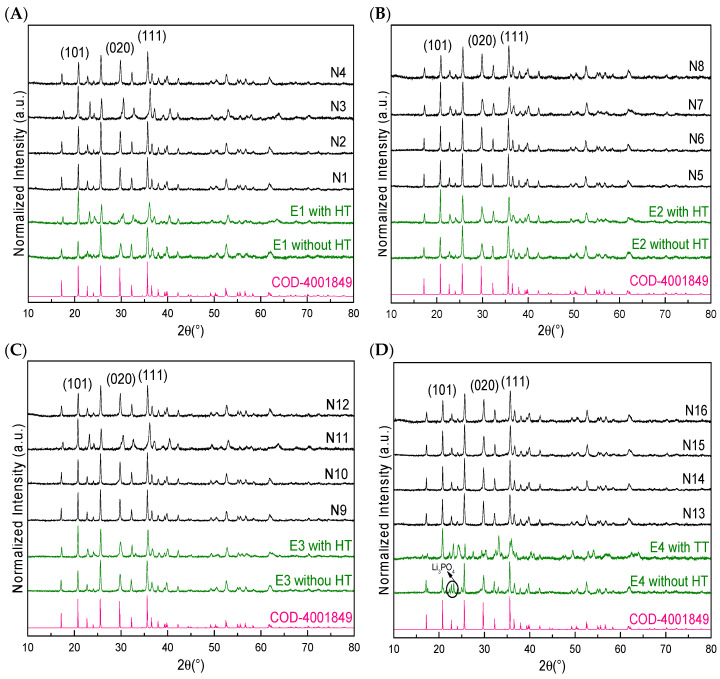
XRD diffractograms of LFP obtained with ethanol before and after the heat treatment (E samples) and with NC (N samples) to form LFP/C composites under different synthesis conditions: (**A**) 150 °C and 15 min; (**B**) 150 °C and 30 min; (**C**) 205 °C and 15 min; (**D**) 205 °C and 30 min.

Figure 1 shows the diffraction peaks of LFP/C composites obtained with different percentages of NC and under different synthesis conditions; for purposes of comparison, compounds without NC and with and without heat treatment (HT) were added (samples E1–E4). The figure shows how the diffraction peak changes according to the synthesis conditions—150 °C and 15 min (Figure 1A), 150 °C and 30 min (Figure 1B), 205 °C and 15 min (Figure 1C), and 205 °C and 30 min (Figure 1D)—and the presence of NC in different amounts (0.15% wt. and 1% wt.). All of the conditions exhibited the diffraction peaks of LFP with an olivine structure and *Pnma* space group [34]; no other peaks of iron species were observed [36], except for sample E4 (Figure 1D), where other diffraction peaks around 22° and 32° were observed and could be attributed to a typical reaction intermediate such as Li_3_PO_4_; however, when NC was used in any form, no other peaks were observed (N samples, Figure 1D).

It can be observed that after the heat treatment of the samples without NC (E samples), the signal of the crystalline phase located at 20.7° was the peak that increased the most in intensity, followed by the signal at 25.6°. This means that after the heat treatment, the crystalline face (101) became preferential, followed by (111). The crystalline face (020), which is the crystalline face responsible for the lithium-ion diffusion [34], increased in intensity when NC was used in the synthesis in all cases but did not increase in the samples without carbon and heat treatment. Therefore, it can be said that the incorporation of NC in the synthesis medium helps to improve the intensity of this crystalline face where the lithium diffusion is located—something that heat treatment does not do.

In general, with the lowest reaction time (Figure 1A,C), low-intensity diffraction peaks of the composites were observed, in contrast to the longer reaction times (Figure 1B,D); the same happened with the E samples (i.e., those without NC). These results show that the reaction time is crucial for obtaining LFP with crystalline and stable behaviour in order to avoid oxidation and the appearance of undesirable diffraction peaks when HT is implemented, independent of the incorporation of NC.

Regarding the NC concentrations, we can usually see a higher intensity with a higher amount of NC. It is possible to notice a decrease and the appearance of unwanted signals close to the lithium-ion diffraction peak (020) located at 29.7° with the sample created with 0.15% wt. of CNF, especially with the shortest reaction time (N3; Figure 1A and N11; Figure 1C), which does not occur with a higher percentage of CNF (N4; Figure 1A and N12; Figure 1C). This difference may be due to the heat treatment used, because the amount of NC incorporated in the composite is carbonised; this process could lead to the LFP material being unrecovered when a low amount is used (like 0.15% wt.), whereas using a higher amount of NC (1% wt.) makes this less likely.

When we compare CNC and CNF, we can observe wider peaks when using CNF rather than CNC; thus, we can expect smaller crystallites with CNF and, consequently, smaller particles. In order to evaluate this parameter, FE-SEM images were taken.

**Figure 2:**
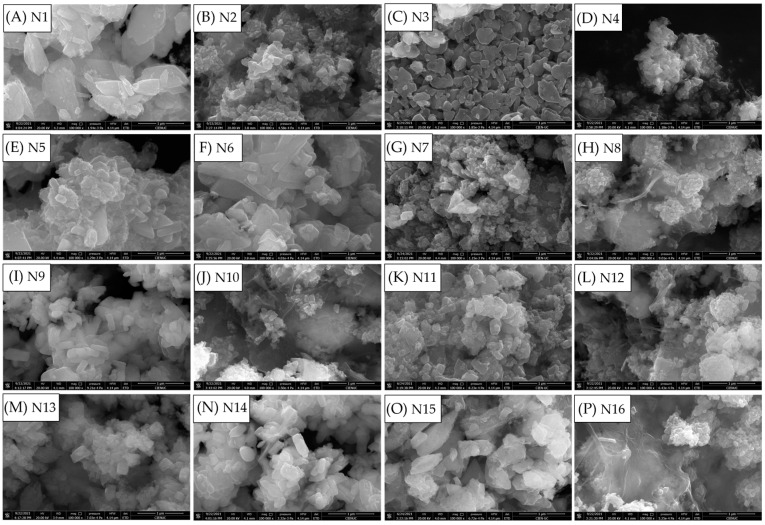
FE–SEM images of LFP/C composites with NC (N samples): (**A**) N1; (**B**) N2; (**C**) N3; (**D**) N4; (**E**) N5; (**F**) N6; (**G**) N7; (**H**) N8; (**I**) N9; (**J**) N10; (**K**) N11; (**L**) N12; (**M**) N13; (**N**) N14; (**O**) N15; (**P**) N16.

Figure 2 shows the FE-SEM images obtained with CNC and CNF (samples N1 to N16). The average particle size was calculated with 200 particles as n-observations, using ImageJ software; Table S1 and Figure S2 show the values obtained and the particle sizes of the E samples for comparisons. In all cases, using CNC or CNF in the synthesis of LFP decreased the average particle size compared to synthesis without NC, demonstrating that the NC acts as an anti-agglomerate agent, helping to create different growing nuclei, as shown in previous studies with other compounds, such as ethylene glycol or urea [37,38]. The particle size differences depend not only on the amount and type of NC, but also on the synthesis conditions of the LFP compound; the largest differences in average particle size were obtained under synthesis conditions involving a longer reaction time (30 min; Figure 2E–H,M–P).

**Figure 3:**
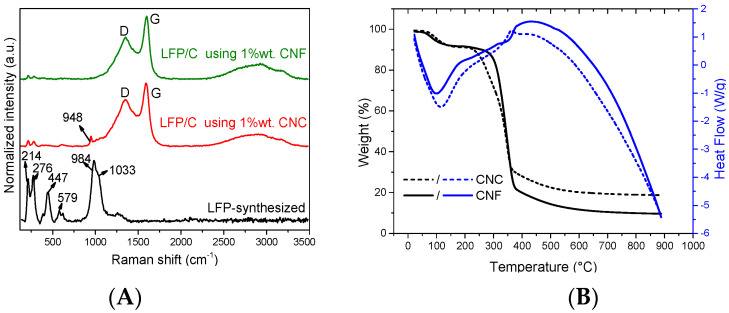
(**A**) Raman spectra of LFP/C composites produced with 1% wt. CNC (red line) and 1% wt. CNF (blue line); LFP synthesised without NC is added for comparison (black line). (**B**) TGA–DSC analysis of CNC and CNF.

In most cases, smaller average particle sizes were obtained when CNF was used in the reaction medium instead of CNC, which is consistent with what was previously seen in the XRD results; hence, the particles obtained using this NC were smaller. Small particle size could be achieved with CNF, because it is known that its fibres are longer than those of CNC [39,40]. Therefore, CNF fibres could avoid agglomeration during hydrothermal synthesis in the microwave vessel better than the shorter CNC fibres. Finally, using a higher amount of NC (1% wt.), smaller particles were obtained; the biggest difference was obtained under the synthesis conditions of 150 °C for 30 min: 147 nm with CNC (Figure 2B) vs. 92 nm with CNF (Figure 3B). This indicates that working with a lower percentage of NC (0.15% wt.) is not sufficient to avoid agglomerations and improve the growth nuclei, leading to larger particle sizes in comparison with when a higher percentage of NC is used. Because of this, it is better to work with 1% wt. NC in the microwave vessel instead of lower percentages; on the other hand, the synthesis conditions of 150 °C for 30 min are the most promising for obtaining LFP, due to the better cell occupancy values with Rietveld refinement (Table S2), since this is enough time for the crystalline structure to be rearranged (unlike the 15 min synthesis condition).

To characterise the carbon obtained in the LFP/C composites, RAMAN spectroscopy was performed on the samples with higher % wt. NC (Figure 3). It can be observed that the typical D and G signals associated with defects in carbon species were observed only in composite materials, and not with the LFP synthesised without NC, indicating the presence of carbon derived only from NC in the synthesis.

The vibrations at 950, 1000, and 1070 cm^−1^ correspond to the three characteristic bands of the phosphate group reported previously in LFP [24], which were not observed in the LFP compound synthesised by the proposed route. Instead, two vibrations were present at 984 and 1033 cm^−1^, which were attributed to the decomposition of LiFePO_4_ into γ-Li_3_Fe_2_(PO_4_)_3_, and the bands at 214 and 276 cm^−1^ were attributed to the oxidation of the compound to α-Fe_2_O_3_ as a result of the laser intensity used in the Raman technique [21,41,42]. These vibrations were weakly observed in the composites formed with both CNC and CNF, confirming that the use of a carbon coating on the LFP particles protects against the decomposition or oxidation of the materials. This can also be confirmed by detecting the 948 cm^−1^ vibration of the sample with CNC (Figure 3A; red line), because this band corresponds to the characteristic bands of the phosphate group in LFP.

To evaluate the quality of the carbon obtained in the composite, the ratios of the D- and G-band intensities (I_D_/I_G_) were measured, where the D band is attributed to disordered carbon bands and the G band to graphitic carbon [43]. A low band ratio is expected to be beneficial for good LFP cathode performance [44], as it would mean that there are more graphitic species (G bands) than amorphous species (D bands). The ratio values for CNF and CNC were 0.72 and 0.84, respectively; therefore, with CNF, we can obtain major graphitic species due to the carbon derived from NC starting to decay during thermal treatment, leaving the material unprotected. Thus, as CNC fibres are shorter than CNF fibres, the graphitic species of the composite decline more significantly in CNC compared to CNF. These results confirm that CNF is a better option for obtaining LFP/C composites with smaller particle size and major graphitic carbon species.

TGA–DSC analysis in a nitrogen atmosphere was carried out for both NCs (Figure 3B) to understand the heat treatment that was used to carbonise them. It was observed that both NCs had the same pattern: a weight loss between 40 and 250 °C that could be attributed to internal water evaporation following glass transition, due to its endothermic behaviour in DSC [45,46]. A second weight loss took place between 250 and 450 °C for the carbonisation of NC into carbon compounds, followed by a continued weight loss due to the decomposition of the carbon material, indicating that carbonisation at 600 °C causes a continuous decomposition of carbon to occur, validating the results of the RAMAN and XDR analyses.

In order to determine the percentage of carbonised carbon after the thermal treatment in the LFP/C powder composites created in the 150 °C and 30 min reaction, an elemental analysis was carried out. Table 2 shows the weight percentages used in the microwave vessel to form the LFP material, along with the weight percentages of carbon obtained in the final LFP/C powder composites.

**Figure 4:**
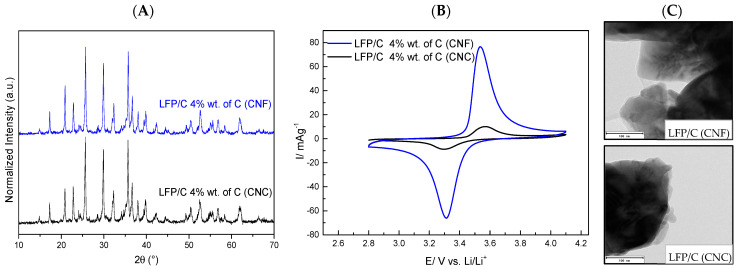
(**A**) XDR of both composites. (**B**) Cyclic voltammetry of LFP/C composites obtained with CNC and CNF at the same weight percentage of carbon (4 % wt.) in LFP/C, measured at 0.1 mV s^−1^ between 2.8 and 4.1 V versus Li/Li^+^ in 1 mol L^−1^ LiPF_6_ with EC/DMC 1:1 *v*/*v*, using the coin cell CR–2032. (**C**) TEM images of LFP/C composites created with CNF (above) and CNC (below).

The carbon obtained after heat treatment was 6.8% wt. with CNC and 12.6% wt. with CNF, confirming that CNC decomposes faster than CNF due to its shorter fibres [47]. This difference in carbon also explains the variation in particle size observed: 369 nm with CNC and 95 nm with CNF, because having less carbon in the composite could lead to LFP particles increasing their size faster than with a higher amount of carbon in the composite, indicating that CNF is a better material to be used for obtaining LFP compounds. In order to confirm this, an equal percentage of carbon (4% wt.) in an LFP/C powder composite was synthesised. Figure 4A shows the diffractograms of both composites, with peaks of only LFP, proving the presence of an olivine structure with the space group *Pnma* formed with the same percentage of carbon (4% wt. C).

Then, an electrochemical characterisation by cyclic voltammetry (CV) was carried out to estimate their performance as cathodes for lithium-ion batteries. Figure 4B shows the typical oxidation peak at 3.5 V and reduction peak at 3.3 V, indicating the presence of LFP in both samples. However, higher current density per weight was obtained in LFP/C composites using CNF as a carbon source (Figure 4B, blue line), which would indicate that the large fibres of CNF could recover the LFP particles better than the short fibres of CNC. The TEM images (Figure 4C) show that a more homogeneous coating is observed using CNF, which is consistent with the cyclic voltammetry results.

**Figure 5:**
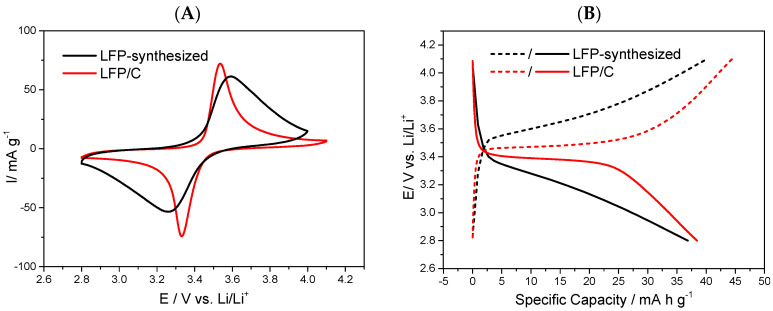
(**A**) Cyclic voltammetry of an LFP/C composite obtained with 12.6% wt. carbon derived from CNF and LFP synthesised without NC, measured at 0.1 mV s^−1^ between 2.8 and 4.1 V versus Li/Li^+^ in 1 mol L^−1^ LiPF_6_ with EC/DMC 1:1 *v*/*v*; second cycle. (**B**) Charge–discharge curve at 0.1 C–rate between 2.8 and 4.1 V versus Li/Li^+^ using the coin cell CR–2032; second cycle.

Finally, the performance of an LFP/C composite obtained with 1% wt. CNF (12.6% wt. final carbon) was evaluated. Figure 5A shows the cyclic voltammetry (black line) of the composite and of LFP synthesised without NC (i.e., carbon; red line). An improvement in electrochemical response and current density per weight can be observed in the composite material as a result of the incorporation of carbon from the NC carbonisation process, enhancing the reversibility of the LFP electrode. Electrical conductivity was measured using a four-point conductivity measurement of prefabricated electrodes of each compound (i.e., synthesised LFP and LFP/C composite), formed as follows: 80% wt. LFP or LFP/C, 10% PVDF, and 10% carbon Vulcan XC-72C, obtaining results of 11.7 kS/m and 49.9 kS/m for the synthesised LFP and the LFP/C composite, respectively, demonstrating that the incorporation of carbon improves the electrical conductivity and enhances the electrochemical performance of this material.

The charge–discharge curves at 0.1 C–rate were derived for the LFP/C composite and the LFP synthesised without carbon materials to evaluate their performance as cathodes. Better behaviour and specific capacity were obtained in the proposed composite compared with LFP without the incorporation of NC during its synthesis, indicating that is possible to obtain a better performance of LFP via the synthetic route in this work compared with LFP synthesised without the incorporation of NC.

The specific capacity of the proposed LFP/C composite was near 40 mAhg^−1^ at 0.1 C, indicating that an improvement of the carbon coating must be investigated to achieve better capacities; however, the incorporation of carbon derived from NC during its synthesis improved its response compared with LFP synthesised without this material.

**Figure 6:**
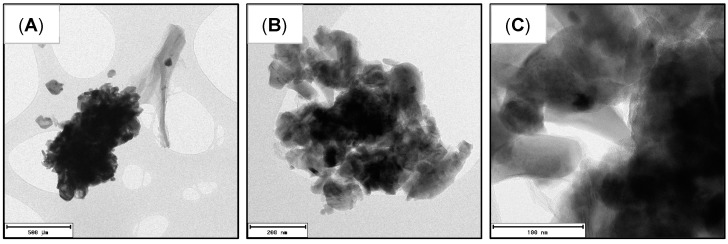
TEM images of composites formed with CNF, at different size scales: (**A**) 500 nm; (**B**) 200 nm and (**C**) 100 nm.

Figure 6 shows different TEM images of the proposed LFP/C composite to evaluate carbon coating and particle size. An accumulation of smaller particles (near 500 nm, Figure 6A), isolated carbon, and covered and uncovered particles can be observed (Figure 6B,C), indicating that NC and LFP particles do not form a homogeneous mixture before heat treatment, so we can observe carbonised NC (conductive carbon) isolated from LFP particles that agglomerate after this heat treatment. Nevertheless, we can also observe particles with a homogeneous carbon coating (Figure 6C).

On the other hand, considering the composite created with 4% wt. carbon (from CNF, Figure 4C), we also observed a homogeneous coating in some particles and less isolated carbon than in the composite created with 12.6% wt. carbon, indicating that an enhancement of the mix of LFP and NC before heat treatment would be beneficial to obtain LFP/C composites through this route with homogeneous coatings of all particles, avoiding excessive amounts of carbon.

### 3.2. X-ray Photoelectron Characterisation of LFP/C Composite

To investigate the material created via the synthetic route, X-ray photoelectron spectroscopy (XPS) characterisation was carried out.

**Figure 7:**
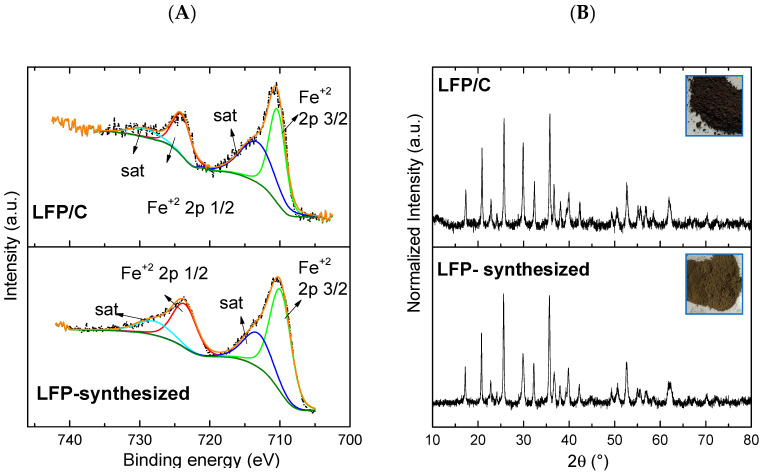
(**A**) High-resolution XPS Fe 2p spectra of synthesised LFP and LFP/C. (**B**) XRD diffractograms of the samples, along with their respective pictures.

Figure 7A shows the high-resolution Fe 2p spectra of the LFP/C composite and of LFP synthesised without carbon (from CNF) as a powder. Through these spectra, it is possible to identify whether the samples contain iron in the 2+ or 3+ oxidation state: signals at 710 eV are attributable to iron 2+ in both samples, and signals near 715 and 729 eV corresponds to satellite peaks that commonly appear in this spectrum when iron 2+ is the predominant species [48,49,50]. Meanwhile, signals attributable to iron 3+ from spin 3/2 that appear at 711–712 eV are not observed. Finally, a cycled coin cell of LFP/C composite for 10 cycles at 0.1 C–rate was investigated in contrast with its cathode without cycling. Thus, an LFP/C composite cathode was opened in a glove box after cycling and then maintained in open-circuit potential to dry the remaining electrolyte, after which it was placed inside the sample holder of the XPS instrument outside the glove box.

**Figure 8:**
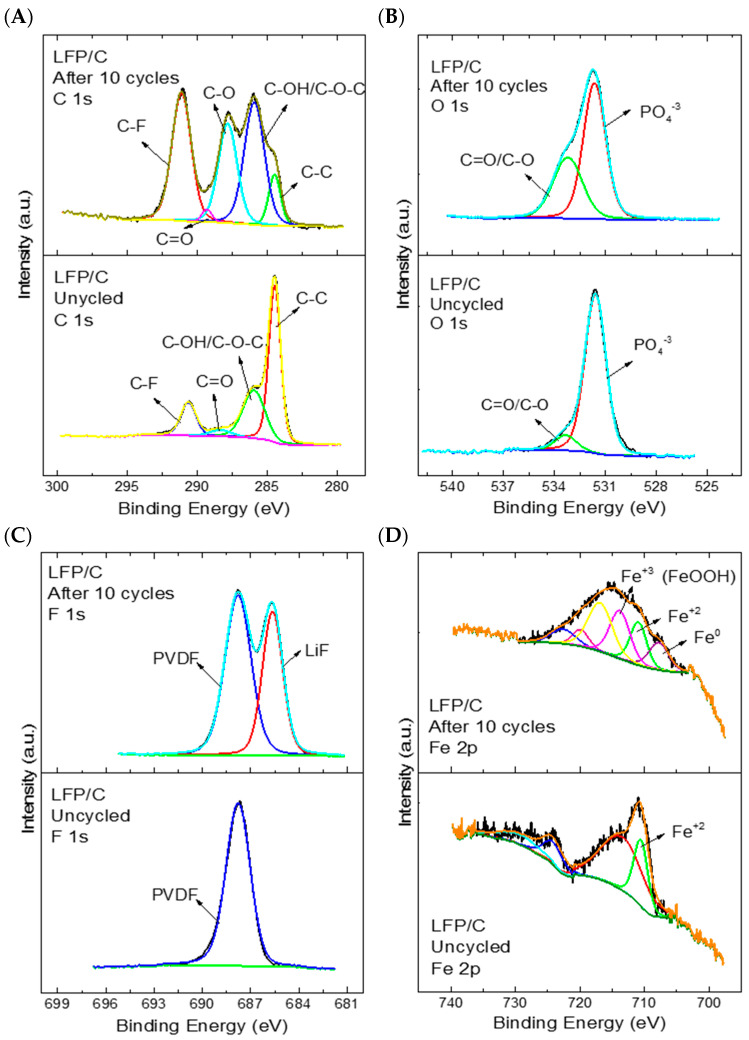
High–resolution XPS spectra of the LFP/C composite before cycling and after cycling for 10 cycles at 0.1 C–rate: (**A**) C 1s spectrum; (**B**) O 1s spectrum; (**C**) F 1s spectrum; (**D**) Fe 2p spectrum.

The C 1s spectrum (Figure 8A) shows the same signals identified in the cathode (below) as in the cycled coin cell (above), but with greater intensity—mainly because the cycled cathode was dried inside the glove box, so it might contain remains of the used electrolyte (LiPF_6_, in EC/DMC solvents) that contain C–C, C–O, and C=O bonds, which would influence the intensities observed in the cathode after cycling. The same tendency can be observed in the high-resolution O 1s spectrum (Figure 8B), with the C–O functional groups; the peak near 531 eV can be attributed to the phosphate group in the LFP material [51]. On the other hand, the appearance of a second signal in the F 1s spectrum (Figure 8C) when the coin cell is cycled indicates the presence of LiF ionic bonds [52] formed for the reduction of anions in carbonate electrolytes [53] (e.g., the LiPF_6_ electrolyte in the EC/DMC solvent that may have remained once the cell was opened to characterise the cathode).

In the high-resolution Fe 2p spectrum (Figure 8D), a clear change in the signals was observed before and after cycling the compound for 10 charge/discharge cycles at a rate of 0.1 C–rate. Before cycling, the iron 2+ species could be clearly identified, but after cycling additional signals appeared that could be attributed to different oxidation states of iron: 0, 2+, and 3+, due to their respective binding energies of 707, 710, and 713 eV. The presence of iron 3+ is expected for the charging and discharging process of a coin cell. The iron changes its oxidation state from 2+ to 3+; therefore, when the cycling is ended and open-circuit potential is achieved, the amounts of iron 2+ and 3+ should be very similar [52,54], since it is not fully charged (Fe^3+^) and not fully discharged (Fe^2+^). These results indicate that the composite loses the reversibility of the Fe^2+^/Fe^3+^ signals after cycling, appearing with other species such as FeOOH or Fe (0) [55], since these elements do not actively participate in the intercalation process of the LFP, which could be the main reason for the specific capacity achieved.

If we compare these results with those of previous works (Table S3), the synthesis conditions reported in this work facilitates obtaining LFP using low temperatures, without unnecessary reagents to prevent iron oxidation (e.g., ascorbic acid), and an in situ carbon coating with organic and sustainable materials such as NC to easily obtain an LFP/C composite. Other synthesis routes use higher temperatures that are detrimental to the synthesis, such as solid-state, ex situ coatings with inorganic and toxic compounds. However, the incorporation of insufficient carbon coating in the LFP/C composite, which improves the reincorporation of lithium ions inside the LiFePO_4_ structure, could lead to iron 3+ or iron 0 species, affecting the specific capacity observed. Therefore, subsequent investigations of homogeneous coatings will be crucial to improve the specific capacity of LFP materials created through this proposed synthesis route.

## 4. Conclusions

When adding NC to the synthesis process, the resulting composites (LFP/C) exhibit better intensity of the crystalline face responsible for lithium diffusion, smaller particle size (with both types of NC), and better electrochemical performance in comparison to LFP synthesised without carbon, due to the carbon coating created by the NC after heat treatment. Between the two NCs used, CNC exhibited better diffraction peaks in XRD than CNF, but we observed wide peaks with CNF, indicating smaller crystallites and, consequently, smaller particles, as evidenced by the FE-SEM images. Comparing the different percentages of NC used, it should be noted that small percentages (i.e., 0.15% wt. NC) can lead to unwanted diffraction peaks due to the possibility of iron oxidation when a small amount of NC is carbonised. In contrast, with a high percentage of NC (i.e., 1% wt.), no unwanted diffraction peaks were observed, but this does not guarantee a proportional decrease in average particle size, and in some cases with CNC an increase in particle size was observed.

Both NCs carbonised to carbon in an inert atmosphere, but through Raman and elemental analysis of the carbon we concluded that CNC decomposes faster than CNF, leading to increase in particle size and a decrease in the final amount of carbon obtained (% wt. carbon in LFP/C composite) when using the same percentage of each NC in the reactor vessel, which is consistent with the results of CV experiments with the same amount of final carbon. As a consequence, the use of CNF instead of CNC in the LFP synthesis shows promising characteristics for use as a cathode: the composite created at 150 °C for 30 min with 1% wt. CNF had 12.6% wt. carbon after heat treatment.

The electrochemical characterisations of the composite showed improved behaviour compared with the LFP synthesised without carbon, confirming that the coating of the LFP particles improves the Li^+^ intercalation behaviour, while a reduced particle size improves the voltammetry profile and the charge–discharge curves.

Through the XPS spectra, it can be seen that the synthesised composite (LFP/C) exhibits only Fe^2+^ species, while after cycling a coin cell of this compound, other iron species such as Fe^3+^ and Fe^0^ can be observed, indicating that inactive species are formed once the coin cell is cycled, which may be responsible for the specific capacity achieved.

Through this proposed synthesis route, we can easily obtain an LFP/C composite, avoiding unnecessary chemicals and multiple steps, and the resultant material shows promise for use as a cathode due to its small particle size (95 nm), carbon coating, and good electrochemical behaviour according to cyclic voltammetry.

Homogeneity of the carbon coating might be the main issue to achieve better capacity, and this should be investigated in further research in order to obtain particles that are fully covered by conductive material.

## Figures and Tables

**Table 1:** Microwave-assisted hydrothermal synthesis conditions of LFP using different types and amounts of NC (CNF and CNC).

Sample	Type NC	%wt. NC	Temperature (°C)	Time (min)
N1	CNC	0.15	150	15
N2	CNC	1	150	15
N3	CNF	0.15	150	15
N4	CNF	1	150	15
N5	CNC	0.15	150	30
N6	CNC	1	150	30
N7	CNF	0.15	150	30
N8	CNF	1	150	30
N9	CNC	0.15	205	15
N10	CNC	1	205	15
N11	CNF	0.15	205	15
N12	CNF	1	205	15
N13	CNC	0.15	205	30
N14	CNC	1	205	30
N15	CNF	0.15	205	30
N16	CNF	1	205	30

**Table 2:** Percentages of NC used in the synthesis, and percentages of carbon obtained after heat treatment in the LFP/C powder composites.

Sample	Type of NC	% wt. of NC Used in Vial Synthesis	% wt. Carbon Obtained in LFP/C Powder
N6	CNC	1	6.8
N8	CNF	1	12.6

## Data Availability

M.K. is the depositary of all the data generated in this study.

## Supplementary Materials

The following supporting information can be downloaded at: https://www.mdpi.com/article/10.3390/polym15122628/s1, Figure S1: FT-IR characterisation of different synthesis conditions of LFP by the microwave-assisted hydrothermal method; Table S1: Average particle size of the N samples, as measured using ImageJ software; Figure S2: FE-SEM images of LFP samples synthesised with ethanol as a reducing agent under different synthesis conditions: (a) 150 °C for 15 min; (b) 150 °C for 30 min; (c) 205 °C for 15 min. The particle distribution is shown as an insert in each image; Table S2: Rietveld values obtained for LFP synthesised using ethanol as a reducing agent, Table S3: Comparison of LFP materials obtained in this study and previous works.
