# Supplementary material for: Lithium Iron Phosphate/Carbon (LFP/C) Composite Using Nanocellulose as a Reducing Agent and Carbon Source
Source: Polymers (Basel). 2023 Jun 9;15(12):2628. doi: 10.3390/polym15122628 (PMC10305069; doi:10.3390/polym15122628)
Supplement: Supplementary file 1 [file polymers-15-02628-s001.zip › polymers-2311089-supplementary.pdf]

## Supplementary Materials

**Figure S1.** FT-IR characterization of different condition synthesis of LFP by microwave-assisted hydrothermal method.

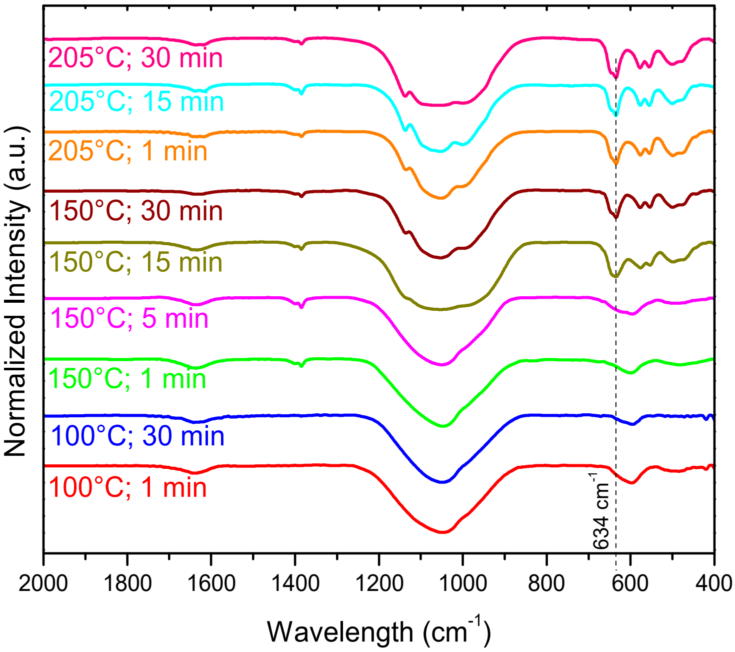


**Table S1.** Average particle size of the N samples, measure with Image J software

| Sample | Type NC | % wt NC | Average particle size (nm) |
|---|---|---|---|
| **N1** | CNC | 0.15 | 369 |
| **N2** | CNC | 1 | 111 |
| **N3** | CNF | 0.15 | 212 |
| **N4** | CNF | 1 | 117 |
| **N5** | CNC | 0.15 | 147 |
| **N6** | CNC | 1 | 369 |
| **N7** | CNF | 0.15 | 92 |
| **N8** | CNF | 1 | 95 |
| **N9** | CNC | 0.15 | 270 |
| **N10** | CNC | 1 | 129 |
| **N11** | CNF | 0.15 | 149 |
| **N12** | CNF | 1 | 108 |
| **N13** | CNC | 0.15 | 159 |
| **N14** | CNC | 1 | 336 |
| **N15** | CNF | 0.15 | 459 |
| **N16** | CNF | 1 | 94 |

**Figure S2.** FE-SEM images of LFP samples synthesized with ethanol as reducing agent at different synthesis conditions.: (a) 150°C for 15 min; (b) 150°C for 30 min; (c) 205°C for 15 min. The particle distribution is inserted in each image.

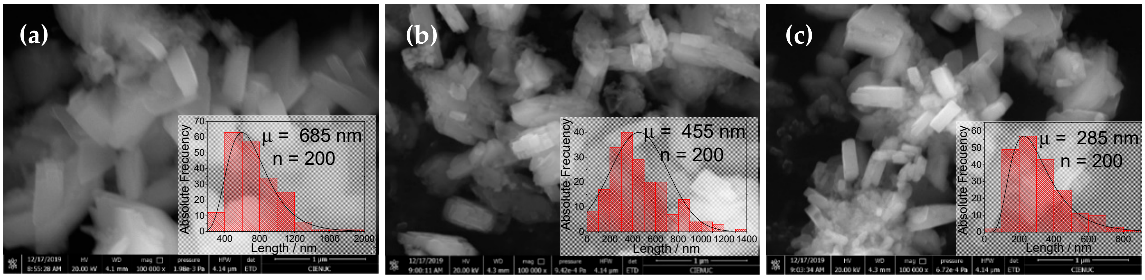


**Table S2**. Rietveld values obtained for LFP synthesized using ethanol as reducing agent.

| Sample | COD-4001849 | LFP-ethanol 150°C and 15 min | LFP-ethanol 150°C and 30 min | LFP-ethanol 205°C and 15 min |
|---|---|---|---|---|
| **a (Å)** | 10.31 | 10.24 | 10.24 | 10.25 |
| **b (Å)** | 6.01 | 5.90 | 5.92 | 5.94 |
| **c (Å)** | 4.69 | 4.66 | 4.67 | 4.66 |
| **Volume (Å$^3$)** | 291.31 | 282.93 | 282.93 | 283.70 |
| **Occ** | Li | 1 | 1 | 1 |
| **Occ Fe** | 1 | 0.456 | 0.873 | 0.677 |
| **Occ P** | 1 | 0.393 | 0.734 | 0.582 |
| **Occ O1** | 1 | 0.475 | 0.998 | 0.785 |
| **Occ O2** | 1 | 0.536 | 0.808 | 0.642 |
| **Occ O3** | 1 | 0.503 | 0.953 | 0.699 |

**Table S3**. Comparison of LFP material obtained in this study and previous works.

| LFP synthesis condition | | | | LFP/C formation | | | Performance | | |
|---|---|---|---|---|---|---|---|---|---|
| **LFP precursors** | **Type of synthesis** | **Temperature and time** | **Reducing agent** | **Carbon source** | **Carbon incorporation** | **Condition** | **Particle size (nm)** | **Specific capacity at 0.1C (mAhg-1)** | **Ref.** |
| LiOH, $FeSO_4$, $H_3PO_4$; naphthenic acid, isooctyl alcohol | Hydrothermal stripping | 250°C; 3 h | Ascorbic acid | No carbon | - | - | ~250 nm | 156,1 | 13 |
| LiOH; $FePO_4$ | Carbothermal reduction | 200°C; 3 h | Glucose | Glucose and synthetic graphite | In situ | Microwave: 1-5 min; 800W | 100-150 | Not informed | 14 |
| $Li_2CO_3$; $FePO_4$ | Solid-state | 650°C; 4 h | Argon | Sucrose | In-situ | - | 46 | 140 | 15 |
| LiOH, $FePO_4$, $H_3PO_4$ | Hydrothermal | 160°C; 12 h | Ascorbic acid | MWCNT | Ex-situ; | 700°C; 6 h in Ar | Non-uniform | 126 | 18 |
| $LiOOCCH_3$, $FeCl_2$, $H_3PO_4$ | Hydrothermal | 160°C; 12h | Ascorbic acid | MWCNT | Ex-situ | - | - | 14 | 21 |
| LiOH, $FePO_4$; $H_3PO_4$ | Hydrothermal, microwave | 150°C, 30 min | NC | CNC or CNF | In-situ | 600°C; 1 h in $N_2$ | 69 | 40 | **This work** |
